# Extracellular Vesicles in Pulmonary Hypertension: A Dangerous Liaison?

**DOI:** 10.3390/biology12081099

**Published:** 2023-08-07

**Authors:** Maria Conti, Marianna Minniti, Mariaenrica Tiné, Miriam De Francesco, Roberta Gaeta, Dario Nieri, Umberto Semenzato, Davide Biondini, Marina Camera, Manuel G. Cosio, Marina Saetta, Alessandro Celi, Erica Bazzan, Tommaso Neri

**Affiliations:** 1 Department of Cardiac, Thoracic, Vascular Sciences and Public Health, University of Padova, 35128 Padova, Italy; maria.conti.2@phd.unipd.it (M.C.); mariaenrica.tine@unipd.it (M.T.); umberto.semenzato@aopd.veneto.it (U.S.); davide.biondini@unipd.it (D.B.); manuel.cosio@mcgill.ca (M.G.C.); marina.saetta@unipd.it (M.S.); erica.bazzan@unipd.it (E.B.); 2 Centro Cardiologico Monzino IRCCS, 20138 Milan, Italy; marina.camera@cardiologicomonzino.it; 3 Centro Dipartimentale di Biologia Cellulare Cardiorespiratoria, Dipartimento di Patologia Chirurgica, Medica, Molecolare e dell’Area Critica, Università Degli Studi di Pisa, 56124 Pisa, Italy; m.minniti2@studenti.unipi.it (M.M.); m.defrancesco3@studenti.unipi.it (M.D.F.); r.gaeta4@studenti.unipi.it (R.G.); dario.nieri@ao-pisa.toscana.it (D.N.); alessandro.celi@unipi.it (A.C.); 4 Department of Medicine, University of Padova, 35128 Padova, Italy; 5 Department of Pharmaceutical Sciences, Università Degli Studi di Milano, 20138 Milan, Italy; 6 Meakins-Christie Laboratories, Respiratory Division, McGill University, Montreal, QC H3A 0G4, Canada

**Keywords:** extracellular vesicles, pulmonary hypertension, pathogenesis, biomarkers

## Abstract

**Simple Summary:**

This review discusses the role of extracellular vesicles in pulmonary hypertension a serious and progressive lung disease characterized by high blood pressure and increased vascular resistance in pulmonary arteries. Extracellular vesicles are released by cells upon different stimuli, contain various molecules like proteins and genetic material derived from the parental cell and are involved in intercellular communication, both for homeostatic processes and in pathologic conditions. They seem to play a harmful role in pulmonary hypertension by carrying and transferring molecules that contribute to the progression of the disease. This review highlights how understanding the mechanisms involving extracellular vesicles in pulmonary hypertension could provide insights into the comprehension of the pathogenesis and in developing new therapies to manage this disease.

**Abstract:**

The term pulmonary hypertension (PH) refers to different conditions, all characterized by increased pressure and resistance in the pulmonary arterial bed. PH has a wide range of causes (essentially, cardiovascular, pulmonary, or connective tissue disorders); however, idiopathic (i.e., without a clear cause) PH exists. This chronic, progressive, and sometimes devastating disease can finally lead to right heart failure and eventually death, through pulmonary vascular remodeling and dysfunction. The exact nature of PH pathophysiology is sometimes still unclear. Extracellular vesicles (EVs), previously known as apoptotic bodies, microvesicles, and exosomes, are small membrane-bound vesicles that are generated by almost all cell types and can be detected in a variety of physiological fluids. EVs are involved in intercellular communication, thus influencing immunological response, inflammation, embryogenesis, aging, and regenerative processes. Indeed, they transport chemokines, cytokines, lipids, RNA and miRNA, and other biologically active molecules. Although the precise functions of EVs are still not fully known, there is mounting evidence that they can play a significant role in the pathophysiology of PH. In this review, after briefly recapping the key stages of PH pathogenesis, we discuss the current evidence on the functions of EVs both as PH biomarkers and potential participants in the distinct pathways of disease progression.

## 1. Introduction

Pulmonary hypertension (PH) is a heterogeneous condition defined by the increase in mean pulmonary arterial pressure, as assessed at right heart catheterization, to values >20 mmHg at rest [1]. Even though it can be the result of different pathways, PH is basically due to pathological remodeling of the vascular bed, which also generates hypercoagulability up to in situ thrombosis. PH is classified in five groups, according to the clinical characteristics and the pathological mechanisms. Among the different types of PH, pulmonary arterial hypertension (PAH) and chronic thromboembolic pulmonary hypertension (CTEPH) usually involve the activation of the coagulation cascade as a key pathobiological mechanism, leading to progressive obstruction of the arterial pulmonary vascular bed and consequently to increased vascular resistance. PAH and CTEPH eventually lead to chronic right heart failure, which has the most relevant impact on morbidity and mortality.

Extracellular vesicles (EVs) are cell-derived membranous structures involved in multiple physiological and pathological processes [2,3]. They are involved in numerous biological pathways, including acute and chronic inflammation and blood coagulation, through the expression of active molecules belonging to the parental cell. EVs have been studied as both biomarkers and pathogenic effectors in PH, and their possible value as therapeutic targets is currently under investigation. This narrative review focuses on the possible role of EVs in PH, with special emphasis on PAH and CTEPH.

## 2. Pulmonary Hypertension: Definition, Classification, Burden, and Clinical Picture

PH is a broad term, encompassing different conditions, all resulting in increased pulmonary arterial pressure and pulmonary vascular resistance.

A pre-capillary and post-capillary form of PH can be distinguished based on the value of the pulmonary artery wedge pressure (PAWP); values of PAWP ≥ 15 mmHg define the post-capillary form [1].

In clinical practice, PH is classified based on the pathogenic mechanisms and the associated or causal conditions and divided in five groups:

- GROUP 1 Pulmonary arterial hypertension (PAH);
- GROUP 2 PH associated with left heart disease;
- GROUP 3 PH associated with lung diseases and/or hypoxia;
- GROUP 4 PH associated with pulmonary artery obstructions (including CTEPH);
- GROUP 5 PH with unclear and/or multifactorial mechanisms.

As previously mentioned, PAH (group 1) is further divided into different subtypes, depending on the possible association with other pathological conditions. The most important entities belonging to group 1 are: idiopathic PAH (IPAH), heritable PAH, PAH associated with connective tissue disease (CTD-PAH) or HIV infection, and PAH associated with drugs and toxins [1].

PH affects approximately 1% of the general population. The prevalence increases in the elderly; indeed, the incidence rises to 10% in individuals over 65 years. In most cases, PH is secondary to left heart disease, while chronic obstructive pulmonary disease is the second leading cause of PH. [4]. When looking at PAH and CTEPH epidemiology, significant differences exist among the different reports. Currently, the majority of robust data comes from both national and non-systematic registries, mainly collected in Western, high-income countries (Europe and United States). There is a wide range in incidence and prevalence of PAH and CTEPH in adults. Indeed, PAH incidence ranges from 1.5 to 32 patients per million (PPM) adults per year, and prevalence from 12.4 to 268 PPM. A similar trend can be seen in CTEPH, whose incidence and prevalence are 0.9–39 per year and 14.5–144 PPM, respectively [5]. In general, prevalence has been increasing in recent years due to the easier access to echocardiograms and right heart catheterization, an ageing population and prolonged survival with current therapeutic approaches [6]. Noteworthy, there is a possible bias in some epidemiological data, depending on the test used for diagnosing or suspecting PH. A study in US population found that the incidence of PAH and CTEPH was about 1.5–2-fold lower when right heart catheterization was considered as the diagnostic criterion, per current guidelines, compared to PH suspected by using echocardiography [7]. Since echocardiography is usually the first step in the PH diagnostic algorithm [1], these data underline the importance of completing the correct diagnostic pathway, in order to not overestimate the burden of the disease [7].

The age at diagnosis of PAH has been rising during the last decades, mostly because of a higher proportion of older patients diagnosed with IPAH, which was once viewed as a disease of the younger age [8]. Currently, US registries show a mean (±standard deviation) age at diagnosis between 50 ± 14 and 65 ± 15 years for PAH [9]. Both PAH and CTEPH are usually most common in women [7,8]. For instance, female sex is a well-known risk factor for scleroderma, which is one of the most important causes of PAH: indeed, 7% to 12% of patients with scleroderma eventually develop PAH [6]. Regarding CTEPH, Italian data showed that from 1% to 8.8% of patients with acute pulmonary embolism develop chronic pulmonary hypertension as a complication, even though in these studies transthoracic echocardiography was mainly used as a diagnostic tool [10]. Another prospective observational study found that CTEPH cumulative incidence was 4% in a median follow-up of 8 years after acute pulmonary embolism. Interestingly, almost all cases of CTEPH were detected within two years from the acute index episode of pulmonary embolism [11].

The typical symptoms of PH are dyspnea (initially only on exertion, but it can eventually develop also at rest), reduced exercise capacity, chest pain, and sometimes syncope. Irrespective of the initial cause, PH almost invariably results in chronic right heart failure, which is a devastating condition associated with a high burden of symptoms and elevate healthcare and social costs, and is mainly responsible for mortality in these patients [12,13].

The advances in the management of PH and the availability of new treatments have considerably improved PH prognosis over the last decades. However, PAH still shows a 3-year survival rate of approximately 70%. In contrast, the prognosis of CTEPH is influenced by the availability of pulmonary thromboendarterectomy: the 3-year survival rate can be as high as 90% after a successful surgery [4].

## 3. Pulmonary Hypertension Pathogenesis

As already said, most cases of PH are due to the advanced stages of common diseases, including chronic heart and respiratory failure (groups 2 and 3) [1]. PAH represents a more challenging entity, characterized by remodeling of pulmonary vessels, increased pulmonary vascular resistance and by right ventricle abnormalities including hypertrophy, chamber dilatation, fat deposition, fibrosis, and metabolic shifts, which eventually result in right heart failure [14]. The small vessels of patients with PAH are characterized by concomitant hypertrophy of endothelial cells, smooth cells and fibroblasts, infiltration of inflammatory cells, and in situ thrombosis [15]. PAH can be associated to rare conditions, including schistosomiasis, HIV, and connective tissue diseases, to specific drugs and toxins, or to specific genetic aberrancies, including the mutations in bone morphogenetic protein receptor 2 (BMPR2), a member of the transforming growth factor β superfamily that accounts for 80% of familial PAH [16]. In 50–60% of cases PAH is idiopathic since no environmental or genetic link can be identified [8].

Animal models and pathologic data in humans support a strong role for perivascular inflammation in the initiation and/or progression of PAH and pulmonary vascular remodeling [17]. Along with inflammatory cells that are recruited on site, resident cells within the vascular wall have the potential to sustain and promote inflammation and remodeling. Under hypoxia, pulmonary endothelial cells express adhesive molecules attracting immune cells and produce leptin which inhibits Treg lymphocytes proliferation [18]. Similarly, smooth cells and fibroblasts can switch to a pro-inflammatory phenotype and favor the vicious cycle of vascular remodeling [18]. Group 4 (CTEPH) represents a small but rapidly growing group of patients with PH. In most cases of pulmonary thromboembolism, when properly diagnosed and treated with anticoagulants the emboli resolve and blood flow is restored. However, in a small proportion (0.1–11.8%, depending on the source) [1], a residual clot remains attached to vessel walls, organizes, and fibrotizes, causes endothelial dysfunction and vessel constriction, thus progressively impairing blood flow. Several conditions have been linked to the impaired resolution of pulmonary embolism, mainly genetic and secondary hypercoagulability states, cancer, and fibrinolysis abnormalities. Of interest, the remodeling does not involve only the obstructed vessels. Actually, more or less severe pulmonary microvasculopathy can be found in distal pulmonary arteries not directly involved by acute embolism; still, this mechanism promotes disease progression [19].

## 4. An overview on Extracellular Vesicles

**Figure 1:**
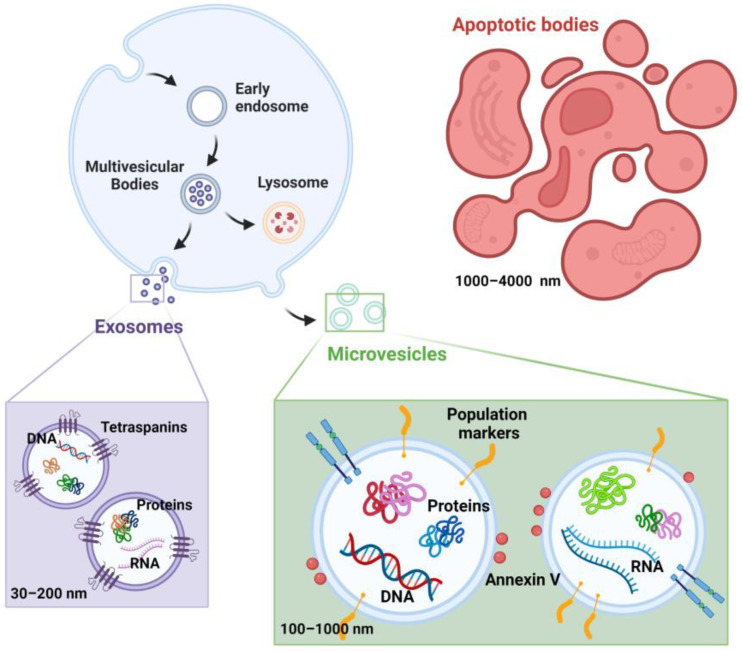
Summary of the EVs (apoptotic bodies, exosomes, and microvesicles) biogenesis, size ranges, surface markers, and cargo. The figure was created through “biorender.com” web site (license agreement number HD25P4J1RM), access date 6 August 2023.

EVs are a family of spherical particles enclosed in a phospholipid bilayer and released by the cell under physiological, such as in brain cell–cell interaction [2] and pathophysiological conditions, such as in COPD, COVID-19, and cardiovascular diseases [3,20,21]. EVs can be detected in different body fluids, such as blood, urine, saliva, bronchoalveolar lavage fluid (BALF), and cerebral and synovial fluids. Due to an increased interest in EVs function and a resulting expansion of scientific publications in the last few years, there is a confusing scientific production concerning EVs classification and nomenclature [22]. According to the MISEV 2018 guidelines [23] these particles can be distinguished in three groups, depending on their biogenesis, the size ranges, and surface markers (Figure 1):

− Exosomes originate from the endo-lysosomal pathway and are identified by the expression of tetraspanins (CD9, CD63, and CD81 among others). They have a dimension between 30–200 nm;
− Microvesicles (also referred to as microparticles or ectosomes) are released by direct outward budding of the plasma membrane of activated cells. They have a dimension between 100–1000 nm and share the same membrane components with the parental cells;
− Apoptotic bodies are released through blebbing of apoptotic cell membranes by cells undergoing apoptosis. Apoptotic bodies have a dimension between 1000–4000 nm.

In practice, the differentiation among the different types of vesicles can prove very difficult; accordingly, the International Society for Extracellular Vesicles suggests the comprehensive term EVs [23]. In this review, we have chosen to maintain the term used by the Authors in the original publications.

The existence and possible functions of EVs were first argued in 1946 by Chargaff while studying platelets and thromboplastin (currently known as tissue factor). He described EVs as cytoplasmic debris [24]. In 1967 Wolf determined by electron microscopy (EM) that normal plasma contained platelet-derived minute particulate material rich in lipid content, responsible for procoagulant activity that he dubbed “platelet dust” [25]. Currently, increasing evidence indicates that EVs also function as carriers for signaling mediators, such as cytokines, inflammatory mediators, and microRNAs (miRNAs), playing a key role for cell-to-cell communication. EVs may contribute to pathogenesis and clinical outcomes of different diseases, such as cancer [26,27], cardiovascular [28,29,30], and pulmonary diseases [31,32,33]. For example, EVs contribute to atherosclerosis progression and plaque rupture promoting microcalcification [34] and are involved in different lung diseases, such as COVID-19 [35] and COPD [3]. In recent years, PH (and particularly PAH) has become a field in which EVs have been actively investigated.

## 5. Extracellular Vesicles as Biomarker and Potential Pathogenetic Effector of PH

EVs have been studied as potential biomarkers for several aspects of both PAH and CTEPH. For example, circulating EVs derived from different cell types are increased in PAH patients [36,37,38,39] and the amount of EVs correlates with functional impairment [38] and mortality [40]. More recently, Kosanovic et al. demonstrated for the first time the augmented T-cell-derived EVs in PH patients suggesting that PAH represents an “inflammatory disorder” in which inflammatory cells are accumulated in the lung and in remodeled pulmonary vasculature [41]. Drug treatment for PAH with prostacyclin analogues (epoprostenol, treprostinil, and iloprost) can inhibit EVs release from platelets and leukocytes [42,43]. A recent study that enrolled 70 patients with PAH shows an increased number of activated platelets- (CD62P+), endothelial- (CD144+) and erythrocytes- (CD235a) derived EVs compared to healthy controls. Of interest, endothelial cell-derived EVs were higher in PAH patients’ urine samples compared to controls and their levels increased with the Tricuspid Annular Plane Systolic Excursion (TAPSE), a key prognostic predictor for PH [44]. In addition, proteomes analysis allowed the identification of 13 proteins differentially expressed, highlighting that EVs from PAH patients contain higher levels of proteins involved in angiogenesis (such as complement C1q and ceruplasmin) [37]. This group showed that the EVs purified from patients with PAH had the potential to provoke surface adhesion markers and oxidative stress—signs of endothelial dysfunction—and to promote pro angiogenic effects on human pulmonary artery endothelial cells in vitro. Another study found that increased levels of endothelial-derived exosomes in IPAH patients has diagnostic potential [45]. Circulating endothelial-derived EVs (EEVs), formerly indicated as microparticles (MPs), have been reported as markers of endothelial injury and systemic vascular remodeling [46] and have been correlated with the degree of endothelial dysfunction in patients with renal and cardiac diseases. PH patients had increased levels of EEVs expressing E-selectin, VE-cadherin and PECAM [37]. PECAM^+^ and VE-cadherin^+^ EVs positively correlate with the mean pulmonary artery pressure (mPAP) and brain natriuretic peptide (BNP) of PH patients, underlying the potential of EEVs as biomarkers of hemodynamic severity of the disease. Indeed, high levels of E-selectin EEVs correlate with poor outcomes or death [40]. A recent study demonstrated the presence of small non-coding RNAs carried by EVs and their possible use as diagnostic and prognostic markers of CTEPH. Among the sncRNAs that were differentially expressed, DQ593939 (a PIWI-interacting RNA-piRNA) correlated with individual clinical parameters (such as mPAP and NT-proBNP) and therefore can be indicated as a potential biomarker for CTEPH [47]. Along with their possible role as biomarkers, EVs have been investigated also as potential actors in PH pathogenesis [43,48,49]. During the development of PAH, a luminal obstruction due to vascular remodeling of the peripheral pulmonary arterial circulation occurs.

Pulmonary arterial endothelial cells (PAECs) present an activated phenotype promoting angiogenesis and recruitment, proliferation, and differentiation of pulmonary arterial smooth muscle cells (PASMCs) through the secretion of growth factors such as transforming growth factor β (TGF-β). Although much progress has been made in the understanding of PAH, much remains to be investigated concerning the molecular mechanisms underlying PAH, particularly the role of EVs in its pathogenesis and their possible therapeutic applications.

**Figure 2:**
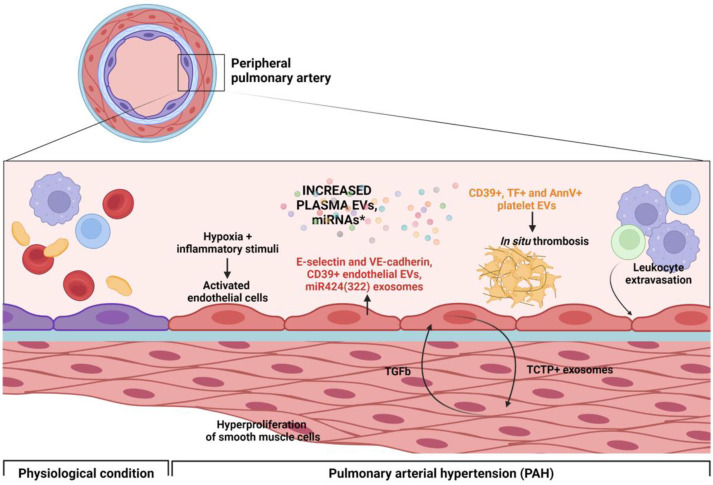
Pathogenesis of pulmonary arterial hypertension (PAH) characterized by remodeling of peripheral pulmonary arterial circulation and a crosstalk between endothelial cells and smooth muscle cells EVs-mediated. *miRNAs: miR-486-5p, miR-26a-5p, miR-596. The figure was created through “biorender.com” web site (license agreement number VQ25OUAYAY), access date 4 August 2023.

Several studies investigated the characteristics and the role of miRNAs in PAH pathogenesis (see Figure 2). miRNAs are small, non-coding RNA molecules found in tissues, plasma and EVs. A recent study compared the miRNA profile contained within purified EVs derived from the plasma of PAH patients and healthy subjects (HS). Among the altered miRNAs, miR-486-5p was overexpressed, while miR-26a-5p was downregulated in PAH EVs [50]. Huang et al. also demonstrated that high levels of plasma exosomal miR-596 are significantly associated with disease severity and poor prognosis of patients with IPAH, despite a lack of investigation of the mechanism of action [51]. miR-424(322), an endothelial cell-specific miRNA that is upregulated due to hypoxia condition, has diagnostic and prognostic value in PAH patients. This miRNA, partially transported in exosomes, acts as the link between the lung and the heart modulating the expression of smad ubiquitination regulatory factor 1 (SMURF1) and contributing to right ventricle hypertrophy and heart failure [52]. An imbalance between cell proliferation and apoptosis is a common feature shared by PAH and cancer, and leads to vascular remodeling and tumor growth, respectively. Translationally controlled tumor protein (TCTP) is known to play a role in proliferation and protection against apoptosis in lung cancer. Lavoie et al. reported an increased TCTP expression in lung tissue sections from patients with PAH and an hyperproliferation of blood outgrowth endothelial cells (BOECs) isolated from patients with PAH overexpressing TCTP [48], suggesting a potential role for TCTP in the hyperproliferation observed in PAH. The protein can be transferred via exosomes between endothelial cells and vascular smooth muscle cells [53]. PAH patients are also characterized by thrombotic lesions and platelet dysfunction, with an alteration of hemostatic and fibrinolytic functions of the endothelium. An increase in CD39 expression and function on platelets and endothelial microparticles in patients with IPAH may contribute to the pathogenesis of IPAH through an increased ATPase and ADPase activity. An alteration of the intravascular nucleotide/nucleoside milieu can indeed affect the vasodilatory and thrombotic response [49]. Elevated plasma levels in von Willebrand factor (vWF), P-selectin and plasminogen activator inhibitor type-1 (PAI-1), and decreased thrombomodulin plasma concentrations were found in PAH patients [54]. Platelet-derived EVs levels are elevated in patients with pulmonary hypertension (both CTEPH and PAH) and, due to their prothrombotic properties, can be involved in the pathogenesis of the disease [43]. Increased pro-coagulant EVs expressing annexin V and tissue factor (TF) were found in the blood drawn from pulmonary arteries of PAH patients and correlate with disease severity. The same authors also identified a population of CD105 (endoglin)-positive EVs that are significantly higher than controls [38]. The data regarding miRNA EVs and their expression in PH are summarized in Table 1.

## 6. Extracellular Vesicles in PH: In Vitro Experiments and Animal Models

Pulmonary EVs derive from various cell types, including lung epithelial cells, macrophages, and pulmonary endothelial cells [60]. Specifically, pulmonary vascular endothelial cells (PVECs) and pulmonary artery smooth muscle cells (PASMCs) have been involved in the pathogenesis of hypoxia-induced PH. Chen et al. demonstrated that the EVs released from PVECs cultured under hypoxic conditions, mostly microvesicles, induced PASMCs proliferation. Furthermore, when PASMCs were administered intravenously to mice models, they induced pulmonary vascular remodeling that led to PH, supporting a switch towards a pro-fibrotic phenotype [61]. Endothelial cells in PH present an “activated” cell phenotype, characterized by modified expression of molecules involved in angiogenesis and local cell growth suppression; they also exhibit disorganized proliferation. Exosomes released by pulmonary artery endothelial cells (PAECs) can promote proliferation while inhibiting apoptosis in PASMCs. This exosome production is controlled by inflammatory and oxidative signaling (LPS and hypoxia) [45]. Zhang et al. have demonstrated an increase in exosomes secretion from PAECs in a mouse model of hypoxia-induced PH. The exosome release inhibitor GW4869 was able to reduce the hypoxia-induced proliferation and migration of PAECs. Moreover, in vivo treatment with GW4869 prevented the dysfunctional and abnormal remodeling of the pulmonary vasculature. Finally, an involvement in the pathogenesis of PH of the lipid-peroxidation enzyme 15-lipoxygenase2 (15LO2) was hypothesized. Notably, 15LO2 was ubiquitinated under hypoxia and further inhibition of the ubiquitin-proteasome system significantly suppressed the proliferation of PAECs. Taken together, all these data suggest that 15-LO2-containing exosomes presumably contribute to the development of PH [62]. Another study investigated the effects of EVs from hypoxic rats on endothelial function and a tissue specificity of the EVs effects in terms of oxidative stress was observed. Overall, the circulating microparticles released under hypoxic conditions induce endothelial dysfunction and are involved in PAH maintenance, primarily by influencing the NO pathway and oxidative stress in the pulmonary vasculature. In particular the authors demonstrated that hypoxic EVs can impair endothelial function in both rat aorta and pulmonary arteries, by directly reducing eNOS activity and by limiting NO bioavailability, and, in the pulmonary bed only, by increasing ROS production [63]. EVs contain various bioactive molecules, including proteins, lipids, and nucleic acids, which can be transferred to recipient cells, thereby influencing their behavior and function. An in vitro study showed that PASMCs can release EVs that alter the normal physiological conditions of PAECs. In summary, de la Cuesta et al. demonstrated that PAECs can uptake PASMC-EVs and efficiently translate their mRNA cargo. These EVs are enriched in Zeb1 and TGF-β superfamily ligands and contribute to endothelial–mesenchymal transition on PAECs during PAH [64]. Another study demonstrated that EC Cav-1 depletion via release of EVs further functions as damage-associated vesicular endothelial signals that stimulate TGF-β–dependent reparative responses by activating and recruiting circulating immune cells. This aspect of the EVs content is interesting because a reduced Cav-1 expression has been reported in different pulmonary inflammatory diseases. During inflammatory vascular conditions, an imbalance in growth factor signaling promotes inhibition of signaling pathways necessary for endothelial cells quiescence and vascular repair, and this may progressively lead to microvascular injury and remodeling as observed in PAH [65]. Moreover, Blair et al. have investigated the impact of selectively circulating EVs on pulmonary artery endothelium in PAH, showing their ability to lead to both surface and intracellular ICAM-1 expression. A selected subset of endoglin+ EVs are responsible for the increase in intracellular ICAM-1 while they do not increase surface expression of the same molecule. Although this is a first preliminary study, it is known that ICAM-1 probably plays an important role in the recruitment of inflammatory cells to pulmonary vascular lesions in PAH. The authors investigated whether circulating EVs from a rat model of severe PAH stimulate localized ICAM-1 on pulmonary endothelium, demonstrating that circulating EVs from late-stage, but not early-stage PAH rats did upregulate ICAM-1 expression on the endothelium and also that ICAM-1 expression was localized to the pulmonary arterial but not to the microvascular endothelium. The finding that late- but not early-stage EVs stimulate an increase in ICAM-1 suggests that the progression of PAH is characterized by a shift in the specific subtypes of EVs and in their biologic effects [66]. Indeed, endoglin, an accessory factor for TGF-β, plays a role in development of pulmonary arterial lesions [67,68]. The data reported in this paragraph are summarized in Table 2.

## 7. Extracellular Vesicles as Potential Therapeutic Targets in PH: Current Evidence and Future Perspectives

Various studies have demonstrated that miRNAs are potential therapeutic targets for several diseases, including PAH. One of the most studied miRNAs is miR-143/145 cluster, which is expressed by vascular smooth muscle cells (VSMCs), and is upregulated in pulmonary artery VSMCs and lung tissue of patients with idiopathic and hereditary PAH [69]. However, the transcriptional regulation of this cluster has not been defined with respect to PAH mediators. Deng et al. have studied the role of this cluster of miRNAs that have recently been found in extracellular compartments, including exosomes [70]. In this study miR-143-3p was upregulated in PAH in animal models as well as in humans. A significant upregulation of miR-143-3p expression was found in mice lung and right ventricle in response to hypoxia. Furthermore, in the primary PASMCs of PAH patients the levels of miR-143-3p and -5p expression were also significantly upregulated compared to the control PASMCs from healthy volunteers [55]. Focusing on EVs as targets of treatment, growing evidence highlights the possible benefits of blocking the release of specific extravesicular miRNAs. When exposed to cigarette smoke, endothelial cells release EVs enriched in miR-1249 which promotes PASMCs hyperproliferation and favors antiapoptotic status [56]. Chronic hypoxia, utilized to induce PH in rats as well in a variety of PH animal models [71], not only provokes hemodynamic and pathologic changes but also increased the number of circulating exosomes and the levels of exosomal miR-211 [57]. When injected in rats, miR-211 overexpressed exosomes could promote PH. Moreover, the group of Aliotta et al. has discovered that PH can be induced in healthy mice by injection of EVs obtained from pulmonary hypertensive mice and that this transfer of disease is mediated by EVs [58]. In a follow-up paper, the same group showed that injection of exosomes but not of microvesicles can induce or reverse monocrotaline-induced PH in mice depending on their source of origin. In particular, exosomes derived from PVECs, which contain increased levels of miRNAs implicated in the pathogenesis of PH, are able to induce PH in mice, whereas exosomes derived from mesenchymal stem cells, which contain miRNAs that induce anti-proliferative, apoptotic, or senescent effects are able to reverse PH in mice [59]. These and other observations pave the way for new therapeutic strategies based on the regulation/modulation of miRNA. Counterbalancing the mainly harmful effect of EVs in PH pathogenesis, some evidence shows a potential protective/reparative mechanism of specific EV subtypes. Belik and colleagues detected an increased amount of endoglin+ endothelial EVs in patients with CTEPH compared to patients with pulmonary embolism and healthy controls [72]. Co-culture of these endoglin+ enriched EVs with endothelial cells improved cell survival and angiogenesis, limiting the effects of vascular occlusion and endothelial damage. On the same line, it has been speculated that the impaired recruitment of pericytes by pulmonary microvascular endothelial cells that contributes to small vessel loss in PAH could be restored by exosomes enriched in Wnt5a, a key signaling factor for pericytes [73]. Moreover, mesenchymal cells collected from bone marrow, adipose tissue, and umbilical cord release EVs that exert several beneficial effects. Preclinical studies have shown that mesenchymal cell derived-EVs have the potential to inhibit the macrophage activation and recruitment that characterize hypoxic PH, reducing the inflammatory infiltrate that eventually promotes pulmonary vascular remodeling [74]. Mesenchymal cell-derived exosomes have been shown to inhibit the hypoxia-induced apoptosis in cultured pulmonary artery endothelial cells and to prevent smooth muscle cell proliferation modulating the Wnt5a/β-catenin pathway [75]. When injected in rats with induced PH, mesenchymal cell-derived EVs have the potential to limit peripheral pulmonary vascular muscularization and reverse the increase in right ventricular systolic pressure and the consequent ventricular wall hypertrophy [76]. Altogether, these data supports that the link between PH and EVs is wide and far from being fully depicted. Indeed, several extravesicular miRNAs have been associated with PH pathogenic mechanisms (summarized in Table 1) and their manipulation could represent a future therapeutic strategy. By restoring the Wnt5a signaling cascade, the delivery of Wnt5a exosomes enriched or mesenchymal cell derived EVs could limit pulmonary vascular remodeling. Further studies on such promising tools and on EVs as therapeutic targets or, themselves, submicroscopic and powerful drugs, broaden our understanding of PH and improve disease management.

## 8. Conclusions

The role of EVs in PH is an area of ongoing research and holds significant promise for understanding the pathogenesis and progression of the disease. EVs released by various cell types involved in PH, such as endothelial cells, smooth muscle cells, and immune cells, have been implicated in vascular dysfunction, inflammation, and remodeling. These EVs can transport bioactive molecules, including microRNAs, proteins, and lipids, which can impact recipient cells in the pulmonary vasculature, influencing vasoconstriction, smooth muscle cell proliferation, and endothelial dysfunction. While our knowledge about EVs in PH is still limited, emerging evidence suggests their involvement in the complex interplay between different cell types and the pathophysiology of PH. Further research is required to uncover the specific cargo and signaling pathways of EVs in different stages of the disease and to determine their potential as diagnostic biomarkers or therapeutic targets. Studying EVs in PH offers a promising avenue for advancing our understanding of this debilitating condition. Continued investigations into the role of EVs in PH may ultimately lead to the development of innovative diagnostic tools and targeted therapeutic interventions that can improve patient outcomes and potentially stop or reverse the progression of pulmonary hypertension.

## Figures and Tables

**Table 1:** Summary of EV size, contents, and expression in PH.

Vesicles Sizes	Contents of Vesicles	Expression in PH	Reference
200–1000 nm	miR-486-5p	Upregulated in PAH EVs	Khandagale et al., 2022 [50]
200–1000 nm	miR-26a-5p	Downregulated in PAH EVs	Khandagale et al., 2022 [50]
30–150 nm	miR-596	Upregulated in IPAH patients	Huang et al., 2021 [51]
100 nm	miR-424(322)	Upregulated due to hypoxia condition in PAH patients	Baptista et al., 2018 [52]
30–130 nm	miR-143-3p and -5p	Upregulated by mice lung and right ventricle in hypoxia PAH	Deng et al., 2015 [55]
100–1000 nm	miR-1249	Upregulated in vitro model of PH	Su et al., 2022 [56]
30–200 nm	miR-211	Upregulated in hypoxia PH rats	Zhang et al., 2021 [57]
30–100 nm	miR-145 and -451	Upregulated in PAH mice model	Aliotta et al., 2013 [58]
30–100 nm	miRs-19b,-20a,-20b, and -145; miRs-34a,-122,-124, and -127	− Upregulated in MCT-injured mice and patients with IPAHU − Upregulated in MSC-exosomes	Aliotta et al., 2016 [59]

**Table 2:** Summary of different EVs effects on PH in in vitro experiments and animal models.

In Vitro Experiments	Target Cells	EVs Origin	Effects	Reference
	PASMCs	PVECs	− EV released by PVECs induce PASMCs proliferation	Chen et al., 2022 [61]
	PASMCs	PAECs	− Exosome released by PAECs promote proliferation/inhibit apoptosis	Zhao et al., 2017 [45]
	PAECs	PAECs	− Hypoxia promote exosome secretion − GW4869 reduce the the hypoxia-induced proliferation and migration of PAECs	Zhang et al., 2018 [62]
	PAECs	PASMCs	− PAECs can uptake PASMC-EVs and translate their mRNA cargo − PASMC-EVs are enriched in Zeb1 and TGF-β superfamily ligands and contribute to endothelial–mesenchymal transition on PAECs during PAH	de la Cuesta et al., 2019 [64]
	ECs	ECs	− Depletion of EC-Cav-1 occurs in part by the release of extracellular Cav-1+ vesicles into circulation and contributes to increased TGF-β signaling, EC proliferation, vascular remodeling, and pulmonary arterial hypertension	Oliveira et al., 2019 [65]
	− PAECs − PMVECs	MPs from rat model of severe PAH	− Late-stage, but not early-stage, MPs selectively induce ICAM-1 in PAECs, but not PMVECs	Blair et al., 2016 [66]
Animal model	Experimental models		Effects	References
	Mouse		− GW4869 prevented the dysfunctional and abnormal remodeling of the pulmonary vasculature − 15-LO2-containing exosomes contribute to the development of PH	Zhang et al., 2018 [62]
	Rat		− Hypoxic EVs impair endothelial function in both rat aorta and pulmonary arteries, by directly reducing eNOS activity and by limiting NO bioavailability, and, in the pulmonary bed only, by increasing ROS production	Tual–Chalo et al., 2010 [63]

## Data Availability

Not applicable.
